# Intrahospital Prevalence of Diabetes and Prediabetes in Medical Departments in Upper Austria

**DOI:** 10.3390/jcm14113668

**Published:** 2025-05-23

**Authors:** Matthias W. Heinzl, Michael Resl, Jörg Kellermair, Clemens Steinwender, Bernhard Mayr, Jana Obereder, Renate Fellner-Färber, Carmen Klammer, Stefanie Hartl, Julia Brandner, Andreas Zierer, David Bernhard, Gersina Rega-Kaun, Julia K. Mader, Michaela Riedl, Harald Stingl, Lars Stechemesser, Claudia Ress, Elke Fröhlich-Reiterer, Johanna M. Brix, Thomas C. Wascher, Harald Sourij, Peter Fasching, Martin Clodi

**Affiliations:** 1 Abteilung für Innere Medizin, Konventhospital Barmherzige Brüder Linz, 4020 Linz, Austria; 2 Clinical Research Institute for Cardiovascular and Metabolic Diseases, Medical Faculty, Johannes Kepler University Linz, 4040 Linz, Austria; 3 Klinik für Kardiologie und Internistische Intensivmedizin, Kepler Universitätsklinikum Linz, 4020 Linz, Austria; 4 Abteilung für Innere Medizin, Salzkammergut-Klinikum Gmunden, 4810 Gmunden, Austria; 5 Universitätsklinik für Herz-, Gefäß- und Thoraxchirurgie, Kepler Universitätsklinikum Linz, 4020 Linz, Austria; 6 Institut für Physiologie und Pathophysiologie, Kepler Universitätsklinikum Linz, 4020 Linz, Austria; 7 5. Medizinische Abteilung mit Endokrinologie, Rheumatologie und Akutgeriatrie, Klinik Ottakring, 1160 Vienna, Austria; 8 Klinische Abteilung für Endokrinologie und Stoffwechsel, Universitäts-Klinik für Innere Medizin, Medizinische Universität Graz, 8036 Graz, Austria; 9 Universitätsklinik für Innere Medizin III, Endokrinologie und Stoffwechsel, Medizinische Universität Wien, 1090 Vienna, Austria; 10 Abteilung für Innere Medizin, Landesklinikum Baden, 2500 Baden, Austria; 11 Uniklinikum Salzburg, Universitätsklinik für Innere Medizin I, 1090 Vienna, Austria; 12 Universitätsklinik für Innere Medizin I, Medizinische Universität Innsbruck, 6020 Innsbruck, Austria; 13 Department für Allgemeine Pädiatrie, Universitätsklinik für Kinder- und Jugendheilkunde, Medizinische Universität Graz, 8010 Graz, Austria; 14 Ist Medical Department, Diabetology, Endocrinology and Nephrology, Klinik Landstraße Wien, 1030 Vienna, Austria; 15 Karl Landsteiner Institute for Obesity and Metabolism, 1030 Vienna, Austria; 16 Medizinische Abteilung, Hanuschkrankenhaus Wien 1, 1140 Vienna, Austria

**Keywords:** prevalence, diabetes, prediabetes, dysglycaemia, intrahospital, HbA1c screening

## Abstract

**Background:** The intrahospital prevalence of diabetes and prediabetes is not well known in Austria and worldwide. Screening for diabetes in hospitalised patients requires systematic glycaemic assessment via HbA1c measurement, which is not routinely performed in all patients in most hospitals. This study is the first multicentre investigation to conduct structured HbA1c screening in hospitalised adult medical patients of all ages. **Methods:** In this exploratory multicentre analysis, HbA1c screening was performed in 3025 consecutive patients hospitalised at three different medical departments in Upper Austria. HbA1c screening was conducted over a period of three months between October 2023 and March 2024. Patients were diagnosed with diabetes (HbA1c ≥6.5% (≥48 mmol/mol)) or prediabetes (HbA1c 5.7–6.4% (39–47 mmol/mol)) based on HbA1c values or a previous diagnosis. **Results:** Dysglycaemia (diabetes or prediabetes) was identified in 1557 patients (51.5%). Diabetes was present in 840 patients (27.8%) and prediabetes in 717 patients (23.7%). A first-time diagnosis of diabetes was made in 73 patients (2.4%). The prevalence of diabetes was highest among patients aged 70–79 years (36.8% diabetes; 24.8% prediabetes). **Conclusions:** Structured HbA1c screening in 3025 consecutive hospitalised patients across three medical departments in Upper Austria revealed a diabetes prevalence of 27.8% and a prediabetes prevalence of 23.7%. Overall, dysglycaemia was present in 51.5% of hospitalised patients.

## 1. Introduction

Diabetes mellitus and prediabetes are associated with increased morbidity and mortality from both vascular and nonvascular conditions, including cancer and infectious diseases, as well as higher hospitalisation rates [1,2,3,4]. Elevated HbA1c is a strong predictor of complications and adverse outcomes, including death and atherosclerotic events [5].

Global prevalence of diabetes mellitus is estimated at 10.5%, with a prevalence of 9.2% in Europe. However, a substantial proportion of undiagnosed cases contributes to uncertainty regarding true prevalence. Furthermore, reported diabetes prevalence varies between studies, largely depending on the method of data acquisition [6,7].

The prevalence of prediabetes is even more uncertain, as it depends on the diagnostic criteria used—impaired fasting glucose (IFG), impaired glucose tolerance (IGT), or HbA1c values between 5.7% and 6.4% [8,9]. According to the IDF Diabetes Atlas, the estimated prevalence of IGT in Europe was 7.1% in 2021 [6]. Globally, the prevalence of IFG and IGT have been estimated at 5.8% and 9.1%, respectively, and these rates are forecast to rise in the coming years, although high-quality country-specific data are lacking in many instances [10].

In Austria, diabetes prevalence in the general population has been reported to range from 5.4% to 9.9%, with prediabetes prevalence estimated at 20.2% by one study, both increasing significantly with age [11,12]. A study from the Salzburg region in Austria reported a mean HbA1c of 5.5% in its study cohort [13]. Epidemiological studies of diabetes in Austria have noted incidence trends that are dependent on age, sex, and location, paralleling broader global trends [14].

Despite the high prevalence and clinical significance of diabetes mellitus, its intrahospital prevalence remains poorly studied. While international guidelines recommend low-threshold screening for diabetes mellitus [8,9], HbA1c measurement is not routinely performed in most hospitalised patients. Most studies reporting diabetes prevalence in hospitalised patients rely on patient history and hospital records with glucose measurements rather than structured HbA1c screening [15]. However, HbA1c measurement is essential for intrahospital diabetes screening since glucose measurements are unreliable for diabetes diagnosis in hospitalised patients due to factors such as stress hyperglycaemia, infections, glucocorticoid administration, and other confounders.

Only very few studies have conducted structured diabetes screening in hospitalised patients, and even those that utilised HbA1c screening often did so in a subset of patients, often those with elevated glucose upon admission or certain age groups, rather than in all patients. These studies reported an intrahospital diabetes prevalence ranging from 12.2% to 29.2% [15,16,17,18,19,20]. Notably, a study from New York that used HbA1c screening reported an intrahospital diabetes prevalence of 50.1% in a community with a high background prevalence of diabetes. However, patients in that study were not included consecutively [21]. A study in maximum care hospitals in Germany found an intrahospital diabetes prevalence of 42.3% in evaluable patients. However, this high prevalence was determined in patients older than 55 years only and diagnosis was based on HbA1c and plasma glucose measurements during hospitalisation [22], thus possibly overestimating the true prevalence. In a German single centre study at a university hospital, retrospective HbA1c measurement revealed prediabetes and diabetes rates of 23.7% and 22.2%, respecively, but enrollment was non-consecutive and HbA1c measurement was only performed in 63.1% of patients [20].

To the best of our knowledge, no previous study has assessed intrahospital diabetes and prediabetes prevalence using prospective structured HbA1c screening across multiple centres in consecutive hospitalised adult patients without age limits. This study aims to determine the prevalence of diabetes mellitus and prediabetes in hospitalised patients through prospective structured screening.

## 2. Methods

### 2.1. Study Sites

This multicentre study aimed to determine the intrahospital prevalence of diabetes mellitus and prediabetes across three medical departments in Upper Austria (Austria, Europe): the Department of Cardiology at the Kepler University Hospital in Linz, the Department of Internal Medicine at the Konventhospital der Barmherzigen Brüder in Linz (Saint John of God Hospital Linz), and the Department of Internal Medicine at the Salzkammergut-Klinikum in Gmunden. These departments serve a diverse population of medical patients, including a very high proportion of emergency admissions. They also provide a balanced representation of urban centres (Linz) and a more rural area (Gmunden), covering a broad spectrum of internal medicine subspecialties. Patients from surgical and other departments were not included.

### 2.2. Study Participants

In total, 3025 consecutive patients admitted to these departments between October 2023 and March 2024 underwent HbA1c screening as part of routine care. Retrospective analysis of data including HbA1c was performed to assess the prevalence of diabetes and prediabetes. Diabetes and prediabetes diagnoses were based on prior medical recordsand HbA1c measurements. In accordance with current guidelines [23,24], diabetes was defined as HbA1c ≥ 6.5% (≥48 mmol/mol) or prior diagnosis, while prediabetes was defined by HbA1c values between 5.7% and 6.4% (39–47 mmol/mol). Occasional inconclusive cases including patients on antidiabetic medication were classified on an individual basis after thorough investigation of prior medical history, but patients were only classified as diabetic if the diagnostic criteria were met unambiguously. Glucose values alone were not considered due to potential confounders. Cardiac disease was defined as coronary artery disease, heart failure, or atrial fibrillation. Atherosclerotic disease was defined as documented peripheral artery disease, cerebrovascular disease, or coronary artery disease.

### 2.3. Ethical Approval

This study was conducted in accordance with the Declaration of Helsinki and International Conference on Harmonization Good Clinical Practice (ICH-GCP) guidelines and was approved by the relevant ethics committee (Ethikkommission der Medizinischen Fakultät, Johannes Kepler Universität Linz, EK Nr: 1032/2023, 22 September 2023). Data were anonymised, and access was restricted to authorised personnel. Patients and members of the public were not actively involved in the design and conduct of this observational study.

### 2.4. Statistics

Data and laboratory values were obtained from hospital records for retrospective analysis. Data collection and analysis were performed using Microsoft^®^ Excel^®^ and IBM^®^ SPSS29^®^. This was an exploratory study. Mann–Whitney U-test and χ^2^-test were applied to test for group differences. For statistical calculations, values below the threshold of detection were set to the threshold of detection.

## 3. Results

### 3.1. General Patient Characteristics

Of the 3025 patients enrolled in the study, 1125 patients (37.2%) were included at the Department of Cardiology, Kepler University Hospital in Linz; 1074 patients (35.5%) at the Department of Internal Medicine, Konventhospital der Barmherzigen Brüder in Linz (Saint John of God Hospital Linz); and 826 patients (27.3%) at the Department of Internal Medicine, Salzkammergut-Klinikum in Gmunden.

**Figure 1:**
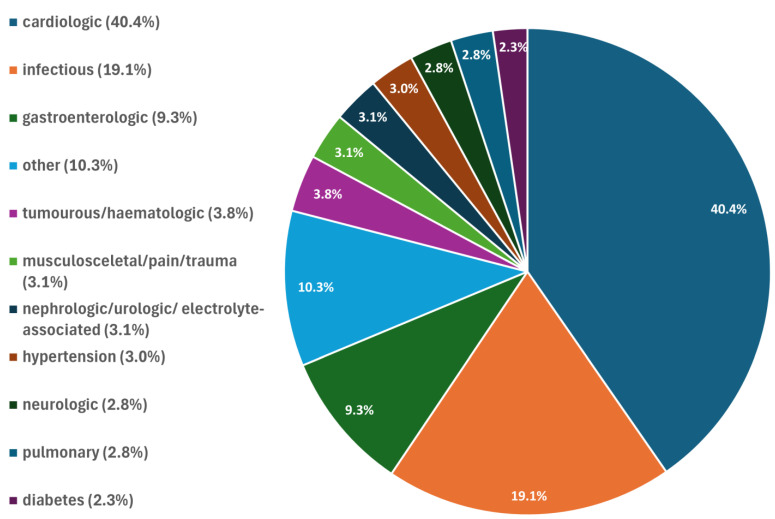
Diagnostic categories of the primary diagnoses for 3025 consecutive hospitalised patients at three medical departments in Upper Austria. Groups of diagnoses were defined arbitrarily and primarily based on medical disciplines and organs. Attribution was performed based on organ systems. Attribution to a group of diagnoses was performed as deemed appropriate by the authors. “other”includes: vascular incl. thrombosis (2.2%); intoxication/substance abuse (1.6%); rheumatologic/autoimmune (0.7%); allergic (0.6%); endocrine (0.2%). “infectious” includes: COVID (2.8%); influenza (1.3%). Infectious diseases and tumours were categorised as infectious and haemato-oncologic conditions, respectively, regardless of the affected organ system.

A total of 986 patients (32.6%) were female, and 2039 (67.4%) were male. The mean age was 70.9 years (SD 15.7), and the median duration of stay was four days (IQR 3–7), as shown in Table 1. A wide range of medical conditions was recorded as the primary diagnosis, as shown in Figure 1.

### 3.2. Diabetes and Prediabetes Prevalence

HbA1c screening was performed in 2787 (92.1%) patients as part of the routine admission procedure, usually on the first working day of hospital stay. Most of the remaining patients who did not undergo HbA1c measurement were admitted and discharged during weekends or holidays, had a short hospital stay, or left the hospital on the day of admission. In the remaining patients without HbA1c measurement, diagnosis of diabetes was only documented if it was known previously.

**Figure 2:**
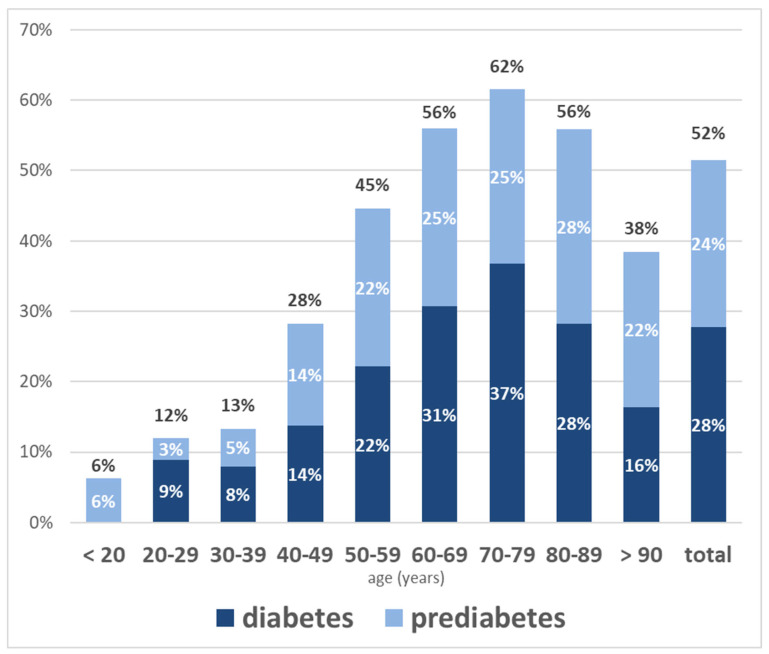
Age-specific intrahospital prevalence of diabetes and prediabetes among 3025 consecutive hospitalised patients at three medical departments in Upper Austria classified according to their HbA1c status. Age groups are shown on the x-axis (age given in years). Columns depict the respective age-specific prevalence of diabetes (dark blue) and prediabetes (light blue), while the total percentage of dysglycaemia (cumulative diabetes and prediabetes prevalence) is shown in black above each column.

Among the 3025 patients, 840 (27.8%) had diabetes, 717 (23.7%) had prediabetes, and 1468 (48.5%) had normal glucose status according to their HbA1c. Thus, dysglycaemia (diabetes or prediabetes) was present in 51.5% of patients. The mean HbA1c overall was 5.9% (SD 1.2%), with values of 7.1% (SD 1.7%) in patients with diabetes, 5.9% (SD 0.3%) in those with prediabetes, and 5.2% (SD 0.3%) in those with normal glucose status. Patients with diabetes had a longer hospital stay, higher BMI, lower eGFR, and were older than those without dysglycaemia, as shown in Table 1. The prevalence of diabetes and prediabetes increased with age, peaking in patients aged 70–79 years (see Figure 2).

The prevalence of diabetes was highest at the Konventhospital der Barmherzigen Brüder in Linz (32.3%), followed by the Department of Cardiology, Kepler University Hospital in Linz (25.8%), and the Department of Internal Medicine, Salzkammergut-Klinikum in Gmunden (24.6%). Prediabetes prevalence was 25.6%, 27.6%, and 16.0%, respectively. These differences were statistically significant (*p* < 0.001, χ^2^-test). The mean BMI was significantly higher at the Department of Cardiology, Kepler University Hospital in Linz (27.3 kg/m^2^) compared to the Konventhospital der Barmherzigen Brüder in Linz (26.3 kg/m^2^, *p* < 0.001, Mann–Whitney U-test) and the Salzkammergut-Klinikum in Gmunden (26.0 kg/m^2^, *p* < 0.001). BMI differences between the latter two institutions were not statistically significant (*p* = 0.352). Details are provided in the Supplementary Table S1.

Among the 840 patients with diabetes, 789 (93.9%) had type 2 diabetes, 35 (4.2%) had type 1 diabetes, two (0.2%) had gestational diabetes, and 14 (1.7%) had diabetes due to non-autoimmune pancreatic disease. Details regarding these patients are provided in the Supplementary Table S2.

Of the 73 patients newly diagnosed with diabetes, one had type 1 diabetes, one had diabetes due to non-autoimmune pancreatic disease, and two had gestational diabetes. The majority of newly diagnosed patients were older than 50 years, with only 11 under 50 years of age. Among these newly diagnosed patients, only 16 had a diabetes-related primary diagnosis. The mean HbA1c was higher in newly diagnosed patients than in those with previously known diabetes (8.0% SD 2.6% vs. 7.1% SD 1.5%; *p* = 0.012, Mann-Whitney U-test).

Mean HbA1c was slightly but significantly lower in female patients than in males (5.9% vs. 6.0%, *p* = 0.002, Mann–Whitney U-test). The prevalence of diabetes was lower in females (22.4%) than in males (30.4%), while prediabetes prevalence was similar (23.8% in females, 23.6% in males). These differences were statistically significant (*p* < 0.001, χ^2^-test). Further gender-based differences are detailed in the Supplementary Table S3.

### 3.3. Relationships Between Glycemic Status and Cardiovascular Illness

As shown in Table 2, the prevalence of cardiac and atherosclerotic disease, as well as arterial hypertension, was significantly higher in patients with dysglycaemia compared to those without. These differences were statistically significant for coronary artery disease, heart failure, atrial fibrillation, atherosclerosis, and cardiac disease (*p* = 0.01 for atrial fibrillation; *p* < 0.001 for all other conditions, χ^2^-test). Differences in cardiovascular disease prevalence were less pronounced between patients with diabetes and those with prediabetes. Arterial hypertension and atherosclerosis were significantly more prevalent in patients with diabetes than in those with prediabetes (*p* = 0.02 and *p* = 0.021, respectively, χ^2^-test). Notably, cardiac disease was more prevalent in patients with prediabetes than in those with diabetes (*p* = 0.03, χ^2^-test).

Conversely, dysglycaemia was more prevalent in patients with cardiac as well as atherosclerotic disease. In patients with heart failure, the prevalence of diabetes and prediabetes was 34.9% and 24.7%, respectively, compared to 25.0% and 22.6% in those without heart failure. In patients with atherosclerotic disease, diabetes and prediabetes prevalence was 34.9% and 26.8%, respectively, compared to 21.5% and 21.0% in those without atherosclerotic disease. More detailed data are available in the Supplementary Table S4.

### 3.4. Relationships Between Glycaemic Status, Medications, BMI, and Mortality

Among patients with diabetes, 85.5% received antidiabetic medication, and 60.7% received lipid-lowering therapy. Medication use is summarised in Table 3. Insulin therapy was initiated in four patients without diabetes for hyperglycaemia correction during hospital stay but was discontinued before discharge.

Among patients with BMI ≥ 30 kg/m^2^, diabetes prevalence was 44.7%, compared to 23.3% in those with BMI < 30 kg/m^2^ (*p* < 0.001, χ^2^-test). Prediabetes prevalence was 24.9% in patients with BMI ≥ 30 kg/m^2^, versus 24.4% in those with BMI < 30 kg/m^2^ (*p* < 0.001, χ^2^-test). There was a significant correlation between HbA1c and BMI (Spearman’s ρ = 0.221, *p* < 0.001). Detailed BMI-stratified data are provided in the Supplementary Table S1.

The overall intrahospital mortality rate was 4.3% (130 patients), with similar rates among patients with diabetes (4.6%), prediabetes (4.5%), and normal glucose status (4.0%) (*p* = 0.753, χ^2^-test). Mortality was also similar in newly diagnosed diabetes patients (4.1%).

In a logistic regression model, HbA1c was not a significant predictor of intrahospital mortality after adjusting for age and BMI (OR = 1.019, *p* = 0.865). Age was the only statistically significant predictor (OR = 1.065, *p* < 0.001). Similarly, HbA1c was not a significant mortality predictor when analysed in patients with dysglycaemia (OR = 0.973, *p* = 0.842) or diabetes only (OR = 0.874, *p* = 0.418).

## 4. Discussion

### 4.1. Prevalence of Diabetes and Prediabetes Prevalence in Hospitalised Patients

In this cohort of 3025 consecutive patients from three medical departments in different hospitals in the cities of Linz and Gmunden (Upper Austria, Austria, Europe), the prevalence of diabetes was 27.8% (840 patients), while the prevalence of prediabetes was 23.7% (717 patients). Consequently, dysglycaemia was present in 51.5% (1557 patients) of the study cohort.

Screening for diabetes among hospitalised patients requires systematic glycaemic assessment via HbA1c measurement. Although HbA1c measurement entails limitations in itself, especially in situations like increased red blood cell turnover, anaemia and haemoglobinopathies, the advantages of HbA1c measurement greatly outweigh its limitations for diabetes screening in hospitalised patients and determination of glucose control [8,9]. Only a limited number of studies have performed diabetes screening in hospitalised patients using HbA1c measurement and have usually done so in a subset of patients only [15,16,17,18,19,20,21,22]. As a result, the prevalence of diabetes mellitus in hospitalised patients remains largely unknown in many regions, and reliable data on the prevalence of prediabetes in this population is lacking, with published studies being single-centre analyses that relied on non-consecutive enrolment [20,21]. This is the first study to conduct structured screening for diabetes and prediabetes using HbA1c measurement in consecutive medical patients of all ages at multiple centres in Austria and, to the best of our knowledge, worldwide.

In most patients with diabetes in our study (767 patients, 91.3% of all diabetes cases), the diagnosis had been established prior to the index hospital admission. In 73 patients (8.7% of all diabetes cases and 2.4% of all patients), diabetes was newly diagnosed. Of these, only 16 had a diabetes-related primary diagnosis, while the remaining 57 were diagnosed with diabetes during hospitalisation for another indication.

A substantial proportion of patients in our study had prediabetes (717 patients, 23.7%), with prevalence increasing with age, as shown in Figure 2 and the Supplementary Tables S5 and S6. Among patients older than 50 years, the prevalence of prediabetes was 25.3%, consistent with higher prediabetes prevalence over the course of aging [11,25], and with rates reported in a prior German study of hospitalized patients [20]. Prediabetes is a highly prevalent condition of significant epidemiological importance, as it increases the risk of progression to type 2 diabetes [26,27], and is associated with a heightened risk of cardiovascular disease, all-cause mortality, chronic kidney disease, and even dementia [27,28,29]. In our study, prediabetes was diagnosed solely based on a prior diagnosis or HbA1c values between 5.7% and 6.4%. The prevalence of prediabetes in hospitalised patients may be even higher if IFG and IGT were also considered [10,25]. However, as discussed above, glucose measurements during hospitalisation are not valid for diagnosing diabetes and prediabetes.

The prevalence of diabetes in our cohort of hospitalised patients was significantly higher than even the highest reported estimates from the Austrian general population, which suggest a prevalence of up to 9.9% [12]. Similarly, the intrahospital prevalence of dysglycaemia (51.5% in our study) was considerably higher than the highest reported prevalence in the Austrian general population (25.6%, based on IFG and HbA1c measurement) [11]. Importantly, the actual number of patients with diabetes may be even higher than indicated by HbA1c screening and prior diagnoses alone, when all parameters including glucose tolerance testing are incorporated [30]. A previous study [31] reported that 23.4% of patients with HbA1c values between 6.0% and 6.4% without previously known diabetes were subsequently diagnosed with diabetes through oral glucose tolerance testing. Applying this finding to our study cohort, at least 52 additional patients could be classified as having diabetes, increasing the total to 892 patients (29.5% of the study cohort).

The risk of hospitalisation is known to be associated with both diabetes and prediabetes and has been shown to correlate with higher HbA1c levels [32], which is reflected by the excess prevalence in hospitalised patients. Additionally, hospitalisations in the preceding year represent a significant risk factor for subsequent hospitalisation in patients with diabetes [33]. Therefore, hospitalisations present an opportunity to implement interventions aimed at improving glucose control and reduce the risk of future admissions, thereby alleviating both the disease burden for patients and the associated socioeconomic costs. Routine HbA1c measurement and screening for diabetes and prediabetes in hospitalised patients, as conducted in our study, appears to be a valuable and easily applicable strategy for identifying patients at an elevated risk, determining disease control in patients with known diabetes, and facilitating timely interventions.

### 4.2. Comorbidities, Antidiabetic Therapy, and Mortality

A high prevalence of atherosclerotic disease (46.8%), cardiac disease (61.9%), and arterial hypertension (56.2%) was observed in the study cohort (differences between centres are shown in the Supplementary Table S7). Notably, these figures only account for known diagnoses in a real-world setting. Especially the true prevalence of atherosclerosis may be considerably higher, as neither peripheral nor cerebrovascular disease was actively screened for in this cohort of patients.

The prevalence of comorbidities, including atherosclerotic and cardiac disease, was markedly higher in patients with diabetes and prediabetes compared to those without dysglycaemia (see Table 2). Conversely, we observed a greater prevalence of diabetes and prediabetes among patients with atherosclerotic and cardiac disease (see Supplementary Table S4). These trends align with the mechanistic overlap thought to mediate the interplay between diabetes and atherosclerotic disease [34]. While the benefits of intensive glucose-lowering therapies are well established in diabetes [35], antidiabetic treatments are currently not commonly used or specifically approved for prediabetes. The high prevalence of prediabetes and its association with comorbidities highlights the clinical significance of this condition and underscores the need for targeted therapeutic interventions.

Our data indicate a high prevalence of heart failure among hospitalised patients both with diabetes and prediabetes compared to those without dysglycaemia (34.6%, 31.0%, and 21.8%, respectively). The true prevalence may be even higher, as heart failure was not systematically screened for in this study cohort. Generally, the risk of heart failure is estimated to be increased by 2- to 4-fold among individuals with prediabetes or diabetes relative to normoglycemic individuals [36,37] and patient collectives in studies of heart failure usually include a very high proportion of patients with diabetes (often up to 50%) [38,39,40,41]. Traditionally, diabetes management has primarily focused on atherosclerotic events and microvascular complications (neuropathy, nephropathy, and retinopathy). However, diabetes and diabetic cardiomyopathy have increasingly been recognised as key contributors to heart failure development [42]. Our findings underscore the importance of diabetes and dysglycaemia as a major factor in the development of heart failure and the complex bidirectional relationship, which involves a broad range of pathophysiological mechanisms [36,43,44].

Among patients with diabetes, 85.5% received antidiabetic medication, with Metformin (50.6%) and SGLT-2 inhibitors (44.9%) being the most commonly prescribed treatments (see Table 3). The number of patients receiving GLP-1 analogues was relatively small (61 patients), and this treatment was initiated in only five cases. This is primarily attributable to the significant shortage of GLP-1 analogues in Europe during the study period [45] as well as a possible incomplete recognition of weekly injectable agents in hospitalised patients. Prior surveys of diabetes incidence in Austria have revealed relatively high rates of medication discontinuation, prompting more general concerns about the need for increased evaluation and monitoring of these patient subpopulations [14].

Mortality rates were comparable between patients with and without diabetes, with an overall mortality of 4.3%. After adjusting for age and BMI in logistic regression, HbA1c was not a significant predictor of mortality (OR = 1.019; *p* = 0.865). However, both patients with diabetes and those with dysglycaemia had significantly longer hospital stays than patients without dysglycaemia (see Table 1). While this link between dysglycaemia and prolonged hospitalization has been noted in some prior studies [46], other teams have also noted increases in in-hospital mortality risk among certain populations of diabetes patients including those who experienced major cardiovascular events or COVID-19 [47,48]. These partially discrepant results may be related to insufficient statistical power, regional variations, or the need to conduct more in-depth analyses based on specific causes of hospitalization.

### 4.3. Strengths and Limitations

This is the first study to determine the prevalence of diabetes and prediabetes in hospitalised patients via HbA1c screening in Austria and, to our knowledge, the first multicentre study to determine the intrahospital prevalence of diabetes and prediabetes through structured HbA1c screening in consecutive adult patients of all ages. Overall, our findings demonstrate an excess prevalence of diabetes and prediabetes in hospitalised patients compared to data from the general population, highlighting the substantial medical and socioeconomic burden associated with these conditions.

Despite these strengths, there are certain limitations to this study. For one, a gender imbalance was present in this study, with 32.6% female and 67.4% male patients. This disproportion primarily reflects the structural organisation of hospital beds in Linz, where the Department of Internal Medicine at the Konventhospital der Barmherzigen Brüder predominantly admits male patients (87.3% male, 12.7% female in this study). The imbalance was less pronounced at the Department of Cardiology of the Kepler University Hospital (62.2% male, 37.8% female), while at the Department of Internal Medicine at the Salzkammergut-Klinikum in Gmunden, slightly more female than male patients were admitted (51.5% female, 48.5% male).

HbA1c screening was performed in 92.1% of all patients only and some patients with unknown diabetes or prediabetes may thus have been missed. Furthermore, the classification of patients into diagnostic groups, as illustrated in Figure 1, is inherently ambiguous. These groups were defined arbitrarily, primarily based on medical disciplines and organ systems. Patient attribution was determined according to the primary diagnosis as deemed appropriate by the authors. Infectious diseases and tumours were categorised as infectious and haemato-oncologic conditions, respectively, regardless of the affected organ system.

Importantly, this study included only patients from medical departments, excluding those from surgical and other specialties. The prevalence of diabetes and prediabetes is strongly dependent on the patient population studied, which is reflected in the differences observed between the three centres. However, this study was conducted across three centres in both urban and rural settings, encompassing departments with different medical specialties and a broad spectrum of medical conditions, thereby accounting for some potential confounders. Patients were included consecutively, and the vast majority were admitted for acute conditions via the emergency department. No patients were electively admitted for antidiabetic treatment, minimising selection bias.

## 5. Conclusions

Among 3025 consecutive hospitalised patients across three medical departments in Upper Austria, the prevalence of diabetes and prediabetes, determined via structured HbA1c screening, was 27.8% and 23.7%, respectively. Thus, dysglycaemia was present in 51.5% of hospitalised patients. Integrating routine HbA1c measurement in hospitalised patients appears to be a valuable and easily applicable strategy for identifying patients with dysglycaemia and determining disease control in patients with diabetes, facilitating timely interventions.

## Figures and Tables

**Table 1:** Characteristics and laboratory values from 3025 consecutive hospitalised patients across three medical departments in Upper Austria classified according to glucose status.

	Diabetes *n* = 840	Prediabetes *n* = 717	Normal Glucose Status *n* = 1468	Total *n* = 3025
	Mean ± SD or Median (IQR)	Mean ± SD or Median (IQR)	Mean ± SD or Median (IQR)	Mean ± SD or Median (IQR)
Duration of stay (days) ^a,b^	5 (3–8)	4 (3–7)	4 (2–7)	4 (3–7)
Age (years) ^a,c^	72.9 ± 11.9	73.8 ± 12.6	68.3 ± 18.4	70.9 ± 15.7
Height (cm)	171.9 ± 8.6	171.7 ± 8.8	171.6 ± 9.3	171.7 ± 9.0
BMI (kg/m^2^) ^a,b,c^	28.4 ± 6.4	26.7 ± 5.2	25.6 ± 4.9	26.6 ± 5.5
HbA1c (%) ^a,b,c^	7.1 ± 1.7	5.9 ± 0.3	5.2 ± 0.3	5.9 ± 1.2
LDL (mg/dL) ^a,b,c^	80.4 ± 39.4	87.8 ± 41.5	98.2 ± 42.3	90.8 ± 42.0
HDL (mg/dL) ^a,b,c^	43.6 ± 17.3	48.0 ± 16.7	51.0 ± 19.9	48.3 ± 18.6
Total cholesterol (mg/dL) ^a,c^	150.3 ± 48.3	154.3 ± 47.8	168.0 ± 47.9	160.3 ± 48.6
Triglycerides (mg/dL) ^a,b^	120.0 (92–164)	104.5 (79–145)	100.0 (76–134)	105.0 (79–142)
eGFR (mL/min/1.73 m²) ^a,c^	62.2 ± 24.7	64.1 ± 22.5	72.1 ± 23.5	67.5 ± 24.0
Creatinine (mg/dL) ^a,c^	1.34 ± 0.80	1.27 ± 0.97	1.14 ± 0.79	1.23 ± 0.84
Haemoglobin (g/dL) ^a,b^	12.8 ± 2.4	13.2 ± 2.2	13.1 ± 2.3	13.1 ± 2.3
CRP (mg/dL)	1.0 (0.2–4.0)	0.6 (0.2–3.4)	0.5 (0.1–3.3)	0.6 (0.1–3.6)

SD = standard deviation. IQR = interquartile range. Data are presented as the mean ± SD, except for duration of stay, triglycerides, and CRP levels, which are presented as the median (IQR) due to skewed distribution. ^a^: Significant difference between diabetes and normal glucose tolerance. ^b^: Significant difference between diabetes and prediabetes. ^c^: Significant difference between prediabetes and normal glucose tolerance. Data were compared using the Mann–Whitney-U-test with Bonferroni correction; *p* < 0.005 was the threshold of significance.

**Table 2:** Characteristics and prevalence of different conditions among 3025 consecutive hospitalised patients at three medical departments in Upper Austria classified according to glucose status.

	Diabetes *n* = 840		Prediabetes *n* = 717		Normal Glucose Status *n* = 1468		Total *n* = 3025	
Female	221	26.3%	235	32.8%	530	36.1%	986	32.6%
Male	619	73.7%	482	67.2%	938	63.9%	2039	67.4%
Coronary artery disease	393	46.8%	332	46.3%	469	31.9%	1194	39.5%
Acute coronary event	36	4.3%	43	6.0%	65	4.4%	144	4.8%
Heart failure	291	34.6%	222	31.0%	320	21.8%	833	27.5%
Atrial fibrillation	273	32.5%	252	35.1%	431	29.4%	956	31.6%
Arterial hypertension	554	66.0%	432	60.3%	713	48.6%	1699	56.2%
Cardiac disease *	563	67.0%	517	72.1%	793	54.0%	1873	61.9%
Atherosclerosis ^§^	494	58.8%	380	53.0%	543	37.0%	1417	46.8%
Intrahospital mortality	39	4.6%	32	4.5%	59	4.0%	130	4.3%

Percentages reflect the proportion of the corresponding column. * Cardiac disease was defined as coronary artery disease, heart failure or atrial fibrillation. ^§^ Atherosclerosis was defined as documented peripheral artery disease, cerebrovascular disease, or coronary artery disease.

**Table 3:** Medication usage among 3025 consecutive hospitalised patients at three medical departments in Upper Austria grouped according to glucose status.

	Diabetes *n* = 840		Prediabetes *n* = 717		Normal Glucose Status *n* = 1468		Total *n* = 3025	
Metformin (preexisting)	378	45.0%	3	0.4%	0	0.0%	381	12.6%
Metformin initiated	47	5.6%	1	0.1%	0	0.0%	48	1.6%
SGLT2-I (preexisting)	302	36.0%	103	14.4%	103	7.0%	508	16.8%
SGLT2-I initiated	75	8.9%	35	4.9%	60	4.1%	170	5.6%
GLP-1-RA (preexisting)	55	6.5%	0	0.0%	1	0.1%	56	1.9%
GLP-1-RA initiated	4	0.5%	0	0.0%	1	0.1%	5	0.2%
DPP-IV-I (preexisting)	201	23.9%	0	0.0%	0	0.0%	201	6.6%
DPP-IV-I initiated	39	4.6%	1	0.1%	0	0.0%	40	1.3%
Antidiabetic medication	718	85.5%	144	20.1%	165	11.2%	1027	34.0%
Pioglitazone (preexisting)	11	1.3%	0	0.0%	0	0.0%	11	0.4%
Pioglitazone initiated	0	0.0%	0	0.0%	0	0.0%	0	0.0%
Insulin (preexisting)	198	23.6%	0	0.0%	0	0.0%	198	6.5%
Insulin initiated	80	9.5%	3	0.4%	1	0.1%	84	2.8%
Statins (preexisting)	440	52.4%	338	47.1%	418	28.5%	1196	39.5%
Statins initiated	62	7.4%	65	9.1%	88	6.0%	215	7.1%
Ezetimibe (preexisting)	137	16.3%	131	18.3%	143	9.7%	411	13.6%
Ezetimibe initiated	54	6.4%	56	7.8%	75	5.1%	185	6.1%
PCSK9-I (preexisting)	2	0.2%	4	0.6%	9	0.6%	15	0.5%
PCSK9-I initiated	1	0.1%	0	0.0%	0	0.0%	1	0.0%
Bemp. Acid (preexisting)	5	0.6%	6	0.8%	9	0.6%	20	0.7%
Bemp. Acid initiated	2	0.2%	1	0.1%	6	0.4%	9	0.3%
Any lipid-lowering med.	510	60.7%	414	57.7%	515	35.1%	1439	47.6%
Aspirin	372	44.3%	306	42.7%	495	33.7%	1173	38.8%
⁠β⁠-blockade	421	50.1%	383	53.4%	598	40.7%	1402	46.3%
RAAS-I	572	68.1%	467	65.1%	698	47.5%	1737	57.4%

Note: insulin therapy was initiated in 4 patients without diabetes to correct high glucose values during hospitalization but was discontinued before discharge. Additionally, 4 patients received Metformin therapy although they never met the diagnostic criteria of diabetes. Percentages reflect the proportion of the respective column. Lipid-lowering medications included the use of statin therapy, bempedoic acid, PCSK9 inhibitors, or ezetimibe. Antidiabetic therapy included the use of metformin, DPP-IV inhibitors, SGLT-2 inhibitors, GLP-1 analogues, pioglitazone, sulfonylurea, or insulin therapy. Bemp. Acid = Bempedoic Acid. med. = medication. -I = inhibitor. -RA = receptor agonist.

## Data Availability

The datasets generated during and analysed during the study are not publicly available due to ethical and privacy reasons but are available from the corresponding author upon reasonable request.

## Supplementary Materials

The following supporting information can be downloaded at: https://www.mdpi.com/article/10.3390/jcm14113668/s1. Supplementary Table S1: Prevalence of diabetes and prediabetes among 3025 consecutive hospitalised patients at three medical departments in Upper Austria classified for BMI. Supplementary Table S2: Characteristics and laboratory values from 3025 consecutive hospitalised patients across three medical departments in Upper Austria classified according to diabetes type. Supplementary Table S3: Differences between genders among 3025 consecutive hospitalised patients at three medical departments in Upper Austria. Supplementary Table S4: Prevalence of diabetes and prediabetes in different conditions among 3025 consecutive hospitalised patients at three medical departments in Upper Austria. Supplementary Table S5: Age-specific prevalence of diabetes (≤50 years, >50 years, and >70 years) among 3025 consecutive hospitalised patients at three medical departments in Upper Austria. Supplementary Table S6: Age-specific prevalence of diabetes and prediabetes among 3025 consecutive hospitalised patients at three medical departments in Upper Austria. Supplementary Table S7: Differences between centres among 3025 consecutive hospitalised patients at three medical departments in Upper Austria.

## Abbreviations

CRP	C-reactive protein
eGFR	Estimated glomerular filtration rate
HbA1c	Glycated haemoglobin A1
HDL	High-density lipoprotein
ICH-GCP	International Conference on Harmonization Good Clinical Practice
IFG	Impaired fasting glucose
IGT	Impaired glucose tolerance
IQR	Interquartile range
LDL	Low-density lipoprotein
OR	Odds ratio
SD	Standard deviation
