# Supplementary material for: Intrahospital Prevalence of Diabetes and Prediabetes in Medical Departments in Upper Austria
Source: J Clin Med. 2025 May 23;14(11):3668. doi: 10.3390/jcm14113668 (PMC12156927; doi:10.3390/jcm14113668)
Supplement: Supplementary file 1 [file jcm-14-03668-s001.zip › jcm-3598566-supplementary.pdf]

Supplementary Appendix

Intrahospital prevalence of diabetes and prediabetes

Supplementary Table S1: Prevalence of diabetes and prediabetes among 3025 consecutive hospitalised patients at three medical departments in Upper Austria classified for BMI

Supplementary Table S2: Characteristics and laboratory values from 3025 consecutive hospitalised patients across three medical departments in Upper Austria classified according to diabetes type

Supplementary Table S3: Differences between genders among 3025 consecutive hospitalised patients at three medical departments in Upper Austria

Supplementary Table S4: Prevalence of diabetes and prediabetes in different conditions among 3025 consecutive hospitalised patients at three medical departments in Upper Austria

Supplementary Table S5: Age-specific prevalence of diabetes (≤ 50 years, > 50 years, and > 70 years) among 3025 consecutive hospitalised patients at three medical departments in Upper Austria

Supplementary Table S6: Age-specific prevalence of diabetes and prediabetes among 3025 consecutive hospitalised patients at three medical departments in Upper Austria

Supplementary Table S7: Differences between centres among 3025 consecutive hospitalised patients at three medical departments in Upper Austria

	BMI < 18.5<br><br>n = 101		BMI<br>18.5-24.9<br><br>n = 1042		BMI<br>25-29.9<br><br>n = 930		BMI<br>30-34.9<br><br>n = 405		BMI<br>35-39.9<br><br>n = 131		BMI ≥ 40<br><br>n = 63	
**Diabetes**	20	**19.8%**	225	**21.6%**	239	**25.7%**	166	**41.0%**	64	**48.9%**	38	**60.3%**
**Prediabetes**	18	**17.8%**	258	**24.8%**	230	**24.7%**	102	**25.2%**	36	**27.5%**	11	**17.5%**
**Normal glucose status**	63	**62.4%**	559	**53.6%**	461	**49.6%**	137	**33.8%**	31	**23.7%**	14	**22.2%**

Supplementary Table S1: Prevalence of diabetes and prediabetes among 3025 consecutive hospitalised patients at three medical departments in Upper Austria classified for BMI

BMI (body mass index) is given in kg/m²

Percentages represent proportions of the corresponding columns

	Type 1 diabetes<br><br>n = 35	Type 2 diabetes<br><br>n = 789	diabetes due to non-autoimmune pancreatic disease<br><br>n = 14	Gestational diabetes<br><br>n = 2	No diabetes<br><br>n = 2185	Total<br><br>n = 3025
	**Mean** ± SD or **Median** (IQR)	**Mean** ± SD or **Median** (IQR)	**Mean** ± SD or **Median** (IQR)	**Mean** ± SD or **Median** (IQR)	**Mean** ± SD or **Median** (IQR)	**Mean** ± SD or **Median** (IQR)
**Duration of stay (days)**	**3** (2-6)	**5** (3-8)	**4** (2-7)	**5** (4-5)	**4** (3-7)	**4** (3-7))
**Age (years)**	**59.6** ± 18.5	**73.8** ± 10.9	**64.2** ± 10.1	**27.5**	**70.1** ± 16.9	**70.9** ± 15.7
**Height (cm)**	**170.8** ±10.7	**172.0** ± 8.5	**171.3** ± 9.8	**160.5**	**171.6** ± 9.1	**171.7** ± 9.0
**BMI (kg/m²)**	**27.4** ± 5.5	**28.6** ± 6.3	**23.5** ± 1.9	**37.9**	**25.9** ± 5.0	**26.6** ± 5.5
**HbA1c (%)**	**8.2** ± 2.0	**7.1** ± 1.6	**7.6** ± 1.8	**5.9**	**5.5** ± 0.4	**5.9** ± 1.2
**LDL (mg/dl)**	**87.8** ± 34.7	**80.0** ± 39.6	**75.2** ± 34.8	**155.8**	**94.3** ± 42.3	**90.8** ± 42.0
**HDL (mg/dl)**	**46.3** ± 12.1	**43.2** ± 17.0	**57.0** ± 38.0	**85**	**49.9** ± 18.8	**48.3** ± 18.6
**Total cholesterol (mg/dl)**	**159.4** ± 37.2	**149.5** ± 48.8	**164.3** ± 22.8	**274**	**163.5** ± 48.3	**160.3** ± 46.6
**Triglycerides (mg/dl)**	**102** (83-126)	**121** (94-166)	**81** (74-121)	**no data**	**101** (77-137)	**105** (79-142)
**eGFR (ml/min/1,73m²)**	**77.1** ± 21.9	**61.2** ± 24.5	**80.2** ± 25.3	**91**	**69.4** ± 23.4	**67.5** ± 24.0
**Creatinine (mg/dl)**	**1.0** ± 0.4	**1.4** ± 0.8	**1.1** ± 0.8	**0.5**	**1.2** ± 0.9	**1.2** ± 0.8
**Haemoglobin (g/dl)**	**13.4** ± 2.0	**12.8** ± 2.5	**11.9** ± 3.0	**10.5**	**13.2** ± 2.3	**13.1** ± 2.3
**CRP (mg/dl)**	**0.5** (0.1-2.9)	**1.0** (0.2-4.0)	**1.7** (0.2-7.9)	**1.0**	**0.5** (0.1-3.4)	**0.6** (0.1-3.6)

Supplementary Table S2: Characteristics and laboratory values from 3025 consecutive hospitalised patients across three medical departments in Upper Austria classified according to diabetes type

SD = standard deviation

IQR = interquartile range

Data are presented as the mean ± SD, except for duration of stay, triglycerides, and CRP levels, which are presented as the median (IQR) due to skewed distribution

	female		male		Total	
	n = 986		n = 2039		n = 3025	
**Diabetes**	221	**22.4%**	619	**30.4%**	840	**27.8%**
**Prediabetes**	235	**23.8%**	482	**23.6%**	717	**23.7%**
**Normal glucose status**	530	**53.8%**	938	**46.0%**	1468	**48.5%**
**First diagnosis of diabetes**	21	**2.1%**	52	**2.6%**	73	**2.4%**
**Coronary artery disease**	315	**31.9%**	879	**43.1%**	1194	**39.5%**
**Acute coronary event**	40	**4.1%**	104	**5.1%**	144	**4.8%**
**Heart failure**	244	**24.7%**	589	**28.9%**	833	**27.5%**
**Atrial fibrillation**	280	**28.4%**	676	**33.2%**	956	**31.6%**
**Arterial hypertension**	604	**61.3%**	1095	**53.7%**	1699	**56.2%**
**Cardiac disease** *	559	**56.7%**	1314	**64.4%**	1873	**61.9%**
**Atherosclerosis** §	375	**38.0%**	1042	**51.1%**	1417	**46.8%**
**Age** (years; mean ± SD)	**72.5** ± 15.9		**70.1** ± 15.6		**70.9** ± 15.7	
**BMI** (kg/m²; mean ± SD)	**26.3** ± 6.0		**26.9** ± 5.3		**26.6** ± 5.5	
**HbA1c** (%; mean ± SD)	**5.9** ± 1.2		**6.0** ± 1.2		**5.9** ± 1.2	

Supplementary Table S3: Differences between genders among 3025 consecutive hospitalised patients at three medical departments in Upper Austria

Percentages represent proportions of the corresponding columns

SD = standard deviation

**Cardiac disease was defined as coronary artery disease, heart failure, or atrial fibrillation*

§Atherosclerosis was defined as documented peripheral artery disease, cerebrovascular disease, or coronary artery disease

	CAD		NO CAD		Acute coronary event		NO Heart Failure		Heart Failure		NO Athero-sclerosis		Athero-sclerosis		NO Cardiac Disease		Cardiac Disease	
**Diabetes**	393	**32.9%**	447	**24.4%**	36	**25.0%**	549	**25.0%**	291	**34.9%**	346	**21.5%**	494	**34.9%**	277	**24.0%**	563	**30.1%**
**Prediabetes**	332	**27.8%**	385	**21.0%**	43	**29.9%**	495	**22.6%**	222	**26.7%**	337	**21.0%**	380	**26.8%**	200	**17.4%**	517	**27.6%**
**Normal glucose status**	469	**39.3%**	999	**54.6%**	65	**45.1%**	1148	**52.4%**	320	**38.4%**	925	**57.5%**	543	**38.3%**	675	**58.6%**	793	**42.3%**

Supplementary Table S4: Prevalence of diabetes and prediabetes in different conditions among 3025 consecutive hospitalised patients at three medical departments in Upper Austria

Percentages represent proportions of the corresponding columns

**Cardiac disease was defined as coronary artery disease, heart failure, or atrial fibrillation*

§Atherosclerosis was defined as documented peripheral artery disease, cerebrovascular disease, or coronary artery disease

	≤ 50 years n = 296		> 50 years n = 2729		> 70 years n = 1832		Total n = 3025	
Diabetes	31	**10.5%**	809	**29.6%**	559	**30.5%**	840	**27.8%**
Prediabetes	27	**9.1%**	690	**25.3%**	472	**25.8%**	717	**23.7%**
Normal glucose status	238	**80.4%**	1230	**45.1%**	801	**43.7%**	1468	**48.5%**

Supplementary Table S5: Age-specific prevalence of diabetes (≤ 50 years, > 50 years, and > 70 years) among 3025 consecutive hospitalised patients at three medical departments in Upper Austria

Percentages represent proportions of the corresponding columns.

Age (years)	≤20 n=16		20-30 n=67		30-40 n=75		40-50 n=138		50-60 n=298		60-70 n=599		70-80 n=775		80-90 n=849		>90 n=208	
Diabetes	0	**0.0%**	6	**9.0%**	6	**8.0%**	19	**13.8%**	66	**22.1%**	184	**30.7%**	285	**36.8%**	240	**28.3%**	34	**16.3%**
Prediabetes	1	**6.3%**	2	**3.0%**	4	**5.3%**	20	**14.5%**	67	**22.5%**	151	**25.2%**	192	**24.8%**	234	**27.6%**	46	**22.1%**
Normal glucose status	0	**0.0%**	6	**9.0%**	6	**8.0%**	19	**13.8%**	66	**22.1%**	184	**30.7%**	285	**36.8%**	240	**28.3%**	34	**16.3%**

Supplementary Table S6: Age-specific prevalence of diabetes and prediabetes among 3025 consecutive hospitalised patients at three medical departments in Upper Austria

Percentages represent proportions of the corresponding columns.

	Saint John of God Linz n = 1074		KUK Cardiology n = 1125		Klinikum Gmunden n = 826		Total n = 3025	
**Diabetes**	347	**32.3%**	290	**25.8%**	203	**24.6%**	840	**27.8%**
**Prediabetes**	275	**25.6%**	310	**27.6%**	132	**16.0%**	717	**23.7%**
**Normal glucose status**	452	**42.1%**	525	**46.7%**	491	**59.4%**	1468	**48.5%**
**First diagnosis of diabetes**	38	**3.5%**	19	**1.7%**	16	**1.9%**	73	**2.4%**
**Female**	136	**12.7%**	425	**37.8%**	425	**51.5%**	986	**32.6%**
**Male**	938	**87.3%**	700	**62.2%**	401	**48.5%**	2039	**67.4%**
**Coronary artery disease**	301	**28.0%**	711	**63.2%**	182	**22.0%**	1194	**39.5%**
**Acute coronary event**	32	**3.0%**	93	**8.3%**	19	**2.3%**	144	**4.8%**
**Heart failure**	255	**23.7%**	353	**31.4%**	225	**27.2%**	833	**27.5%**
**Atrial fibrillation**	306	**28.5%**	433	**38.5%**	217	**26.3%**	956	**31.6%**
**Arterial hypertension**	552	**51.4%**	676	**60.1%**	471	**57.0%**	1699	**56.2%**
**Cardiac disease** *	541	**50.4%**	944	**83.9%**	388	**47.0%**	1873	**61.9%**
**Atherosclerosis** §	423	**39.4%**	744	**66.1%**	250	**30.3%**	1417	**46.8%**
**Age** (years; mean ± SD)	**69.9** ± 17.0		**70.3** ± 13.3		**72.8** ± 16.9		**70.9** ± 15.7	
**BMI** (kg/m²; mean ± SD)	**26.3** ± 5.5		**27.3** ± 5.4		**26.0** ± 5.5		**26.6** ± 5.5	
**HbA1c** (%; mean ± SD)	**6.0** ± 1.3		**5.9** ± 0.9		**5.9** ± 1.5		**5.9** ± 1.2	

Supplementary Table S7: Differences between centres among 3025 consecutive hospitalised patients at three medical departments in Upper Austria

Percentages represent proportions of the corresponding columns

SD = standard deviation

KUK Cardiology = Department of Cardiology at the Kepler University Hospital in Linz

Saint John of God Linz = Department of Internal Medicine at the Konventhospital der Barmherzigen Brüder in Linz

Klinikum Gmunden = Department of Internal Medicine at the Salzkammergut-Klinikum in Gmunden

[*]Cardiac disease was defined as coronary artery disease, heart failure or atrial fibrillation

[§]Atherosclerosis was defined as documented peripheral artery disease, cerebrovascular disease, or coronary artery disease
